# Distinct MEG correlates of conscious experience, perceptual reversals and stabilization during binocular rivalry

**DOI:** 10.1016/j.neuroimage.2014.06.023

**Published:** 2014-10-15

**Authors:** Kristian Sandberg, Gareth Robert Barnes, Bahador Bahrami, Ryota Kanai, Morten Overgaard, Geraint Rees

**Affiliations:** a Cognitive Neuroscience Research Unit, Hammel Rehabilitation and Research Center, Voldbyvej 15, 8450 Hammel, Denmark; b Cognitive Neuroscience Research Unit, Aarhus University Hospital, Noerrebrogade 44, Building 10G, 8000 Aarhus C, Denmark; c Institute of Cognitive Neuroscience, University College London, 17 Queen Square, WC1N 3AR London, United Kingdom; d Wellcome Trust Centre for Neuroimaging, Institute of Neurology, 12 Queen Square, WC1N 3AR London, United Kingdom; e Interacting Minds Centre, Aarhus University, Jens Chr. Skous Vej 4, Building 1483, 3rd floor 8000 Aarhus C, Denmark; f Sackler Centre for Consciousness Science, School of Psychology, Pevensey 1, BN1 9QH Falmer, United Kingdom

**Keywords:** Consciousness, Binocular rivalry, Stabilization, Perceptual reversals, MEG, Magnetoencephalography

## Abstract

During binocular rivalry, visual perception alternates spontaneously between two different monocular images. Such perceptual reversals are slowed or halted if stimuli are presented intermittently with inter-stimulus intervals larger than ~ 400 ms — a phenomenon called stabilization. Often, the neural correlates of reversal and stabilization are studied separately, and both phenomena in turn are studied separately from the neural correlates of conscious perception. To distinguish the neural correlates of perceptual content, stabilization and reversal, we recorded MEG signals associated with each in the same group of healthy humans observing repeated trials of intermittent presentation of a dichoptic stimulus. Perceptual content correlated mainly with modulation of stimulus-specific activity in occipital/temporal areas 150–270 ms after stimulus onset, possibly reflecting inhibition of the neural populations representing the suppressed image. Stability of perception reflected a gradual build-up of this modulation across at least 10 trials and was also, to some extent, associated with parietal activity 40–90 ms and 220–270 ms after stimulus onset. Perceptual reversals, in contrast, were associated with parietal (150–270 ms) and temporal (150–210 ms) activity on the trial before the reversal and a gradual change in perception-specific activity in occipital (150–270 ms) and temporal (220–420 ms) areas across at least 10 trials leading up to a reversal. Mechanistically, these findings suggest that stability of perception during rivalry is maintained by modulation of activity related to the two monocular images, and gradual adaptation of neuronal populations leads to instability that is eventually resolved by signals from parietal and late sensory cortices.

## Highlights

• Neural correlates of perception, reversals and stabilization were examined.
• Stability of perception is maintained by modulation stimulus-specific activity.
• Gradual adaptation of this modulation leads to a state of instability.
• Instability is resolved by parietal/temporal sources causing perceptual reversals.

## Introduction

Perceptually ambiguous stimuli have long been used to study the neural correlates of visual awareness as several different contents of consciousness can be elicited by the same physical stimulus. Binocular rivalry (BR; Blake and Logothetis, 2002, Blake and Wilson, 2011, Breese, 1899, Tong et al., 2006) is a form of bistable perception that occurs when an image is viewed monocularly while at the same time another, incongruent, image is presented to the same retinal location in the other eye. Perception alternates spontaneously between each monocular view every few seconds; but if a blank interval longer than ~ 400 ms is inserted between intermittent periods of binocular presentations, the perceptual alternation rate drops dramatically as a function of the duration of the blank interval (Leopold et al., 2002). Perception during consecutive trials stabilizes to one of the two monocular alternatives implying the existence of a perceptual memory across subsequent trials, a phenomenon termed stabilization (Leopold et al., 2002).

The intermittent presentation paradigm, where bistable stimuli are presented for < 1 s separated by blank intervals of anywhere between 100 ms and 10 s, has been used to study the neural correlates of conscious perception (Sandberg et al., 2013) as well as those of perceptual reversals (see Kornmeier and Bach (2012) for a review) and stabilization (see Pearson and Brascamp (2008) for a review). Although Leopold et al. (2002) recognized the importance of the paradigm for studying changes in conscious perception in general, there is, nevertheless, very little comparison between studies of the neural correlates of conscious perception and of the neural correlates of perceptual reversals/stabilization. Even comparisons between studies of the correlates of reversals and stabilization are infrequent. In one example of cross-referencing Koivisto and Revonsuo (2010) referred to Kornmeier and Bach’s (2004) Reversal Negativity as possible modulation of consciousness-specific activity, and in another example, Pearson and Brascamp (2008) referred to Reversal Positivity as potential evidence for the involvement of early visual areas in stabilization. However, until now there has been no explicit comparison within the same participants of these potentially different neural mechanisms.

In the present experiment, we intensively examined the neural correlates of conscious perception, reversals, and stabilization in the same participants using intermittent presentations of binocular rivalry. The first goal was to map the MEG correlates of perceptual content during binocular rivalry. We then mapped the MEG correlates of stabilization/reversals and examined which of these correlates reflected modulation of percept-specific activity. Finally, we examined the temporal extent of these correlates (i.e. across how many trials before/after a reversal each type of activity was observed). This allowed us to separate components associated with stabilization (which were expected to be found on many trials before/after a reversal) from those more directly associated with the perceptual reversal (i.e. activity specific to the trials immediately before and/or after the reversal).

## Materials and methods

Data analyzed in the present article were previously reported in Sandberg et al. (2013). This previous article was concerned only with the correlates of conscious perception.

### Participants

Eight healthy young adults (six females; 21–32 years mean 26.0 SD 3.55)) with normal or corrected-to-normal vision gave informed consent to participate in the experiment, which was approved by the UCL Research Ethics Committee.

### Apparatus and MEG recording

Stimuli were generated using the MATLAB toolbox Cogent (http://www.vislab.ucl.ac.uk/Cogent/). They were projected onto a 19” screen at a resolution of 1024 × 768 pixels and at a refresh rate of 60 Hz using a JVC D-ILA, DLA-SX21 projector. Participants viewed the stimuli through a mirror stereoscope positioned at approximately 50 cm from the screen. MEG data were recorded in a magnetically shielded room with a 275 channel CTF Omega whole-head gradiometer system (VSM MedTech, Coquitlam, BC, Canada) at a 600 Hz sampling rate. After participants were comfortably seated, head localizer coils were attached to the nasion and 1 cm anterior of the left and right outer canthus to monitor head movement during the recording sessions.

### Stimuli

**Fig. 1:**
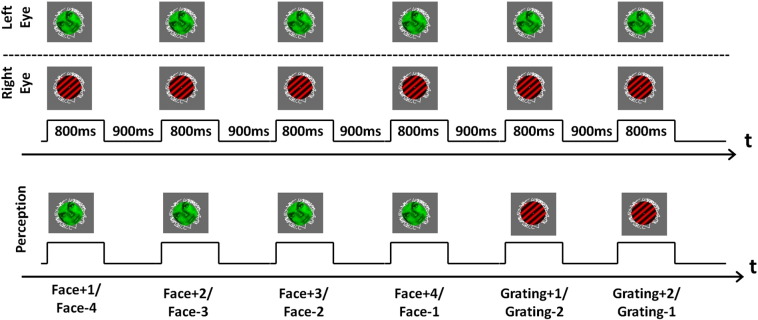
Experimental setup. *Top*: Stimuli (a face and a grating) were presented dichoptically to the eyes of the participant. Stimuli were counter-rotating at a rate of 0.7 rotations per second. Trials were ~ 800 ms and the inter-trial interval was around ~ 900 ms. *Bottom*: Participants reported their perceptual experience (face/grating/mixed perception) with a button press as soon as they were able to meaningfully categorize the trial. Trials were labeled according to their position in relation to a perceptual reversal (for simplicity, all trials after the 10th in a series of identical perceptual reports were assigned the label 10). For instance, the first of four successive trials of face perception was labeled both “Face + 1” (as it was the first perceived face after a series of gratings or mixed percepts) and “Face − 4” (as it was the fourth face perception trial before a perceptual reversal). These labels are used as weights in the analyses below and are referred to as stabilization weights and destabilization weights respectively.

A red Gabor patch (contrast = 100 %, spatial frequency = 3 cycles/degree, standard deviation of the Gaussian envelope = 10 pixels) was presented to the right eye of the participants, and a green face was presented to the left eye (Fig. 1). To avoid piecemeal rivalry, the stimuli rotated at a rate of 0.7 cycles per second in opposite directions, and in order to prevent the stimuli from being perceived in non-overlapping areas of the visual field, each stimulus was presented within an annulus (inner/outer r = 1.3/1.6 degrees of visual angle) consisting of randomly oriented lines. In the center of the circle was a small circular fixation dot.

### Procedure

Participants looked into the mirror stereoscope while the fixation circles around the stimuli were displayed, and the stereoscope was calibrated by adjusting the mirrors until the circles fused. In order to minimize perceptual bias (Carter and Cavanagh, 2007), we increased the chances that participants would report each percept equally often during the experiment by adjusting the relative luminance of the images for each participant before the experiment. The starting luminance for each image was maximum screen value, and one value was decreased until the participant reported seeing both images equally often (+/− 5%) during a one minute long continuous presentation. For all participants, the luminance of the green face was decreased, and the end luminance (used in the experiment) was 58.0% (SD = 17.9%) of maximum screen value. Physical luminance was not measured. When stimuli were displayed during the calibration phase, participants reported what they saw using one of three buttons, each corresponding to either face, grating, or mixed perception. During the experiment, participants used the same three report options but swapped the hand used to report between blocks in order to minimize report-related confounds.

Each participant completed 6–9 runs consisting of 12 identical blocks of 20 identical trials, i.e. a total of 1440–2160 trials were completed per participant. Each trial consisted of presentation of the rivalling images (face/grating), and trials were separated by a blank (grey) screen. The exact durations of stimulus and blank presentation were calibrated for each participant for the reasons given in the following. Immediately after onset of binocular rivalry, participants may perceive a mix of the two images for around 150 ms before one image is perceived clearly (O’Shea and Crassini, 1984, Wolfe, 1983). As it was of key importance that participants were able to distinguish mixed perception throughout a trial from any initial mixed perception (which they were told to not report), stimulus duration had to be longer than a few hundred milliseconds. However, for longer stimulus durations, there is a risk that a reversal may occur during the presentation, and we wanted to avoid this as it would have an impact on the perceptual decisions of the participants and the interpretation of the study. The exact stimulation period was thus calibrated individually for each participant so that they experienced a stable perceptual state, i.e. perception had time to form and did not switch during the stimulation period. This resulted in stimulus durations of 750–900 ms (mean = 806, SD = 50.0) across participants. Furthermore, the duration of the blank periods has a large impact on the degree of stabilization (Orbach et al., 1963). In order to ensure that participants experienced a high degree of stabilization (but not 100%), each trial was separated by a blank (grey) screen appearing for around 800–1000 ms (mean = 931, SD = 80.0) (Fig. 1). Between each block participants were given a short break of 8 s. Between runs participants took a break and signaled when they were ready to start the next run. Participants were instructed not to blink outside of the breaks between blocks and were generally able to follow these instructions. They were further instructed that if they needed to blink outside of the breaks, they should do it after stimulus offset.

### Preprocessing

SPM8 (http://www.fil.ion.ucl.ac.uk/spm/) was used for preprocessing the data. Before analysis, all datasets of the individual runs of each participant were high-pass filtered at 0.5 Hz and downsampled to 300Hz.^1^ Next, the data were epoched from − 600 to 1400 ms around stimulus onset, and the reports of perception were used to divide stimulation intervals into face (44.1%, SD = 13.8), grating (38.6%, SD = 15.2) and mixed epochs (17.3%, SD = 13.0). Trials with mixed perception were not analyzed and were thus only used to establish when stabilization no longer occurred. Trials were then relabeled based on the behavioral responses: the first reported face after a series of grating or mixed perception was thus labeled “Face + 1”, the second “Face + 2”, and so on up to a maximum of 10 at which point perception was presumed to be fully stable. This subdivision allowed us to examine the modulations of the MEG signal as a function face stabilization (using linear models based on Face 1–10 trials), grating stabilization (using linear models based on Grating 1–10 trials), general stabilization (using linear models based on Face 1–10 and Grating 1–10 trials) and perception (contrasting face and grating perception using all trials of each kind). When examining destabilization (i.e. signal changes prior to a perceptual switch), data were labeled so that “Face − 1” was the last trial of reported face perception before a perceptual reversal, “Face − 2” the trial before that and so on. Finally, before source reconstruction, trials containing artifacts were removed at a threshold of 3pT – on average 1.06% (SD = 1.46) – and the data were low-pass filtered at 30 Hz. Visual inspection of the data revealed that the instructions on when to blink (see “Procedure”) were generally followed and eye blinks were generally not observed in the analyzed epochs. Furthermore, as all analyses were performed on data reconstructed in source space (see below), activity related to eye blinks/movement is localized to the cortical surface around the eyes, and activity at these sources within the analyzed epoch was insignificant and not included in the reconstructed dataset.

### Data analysis

**Fig. 2:**
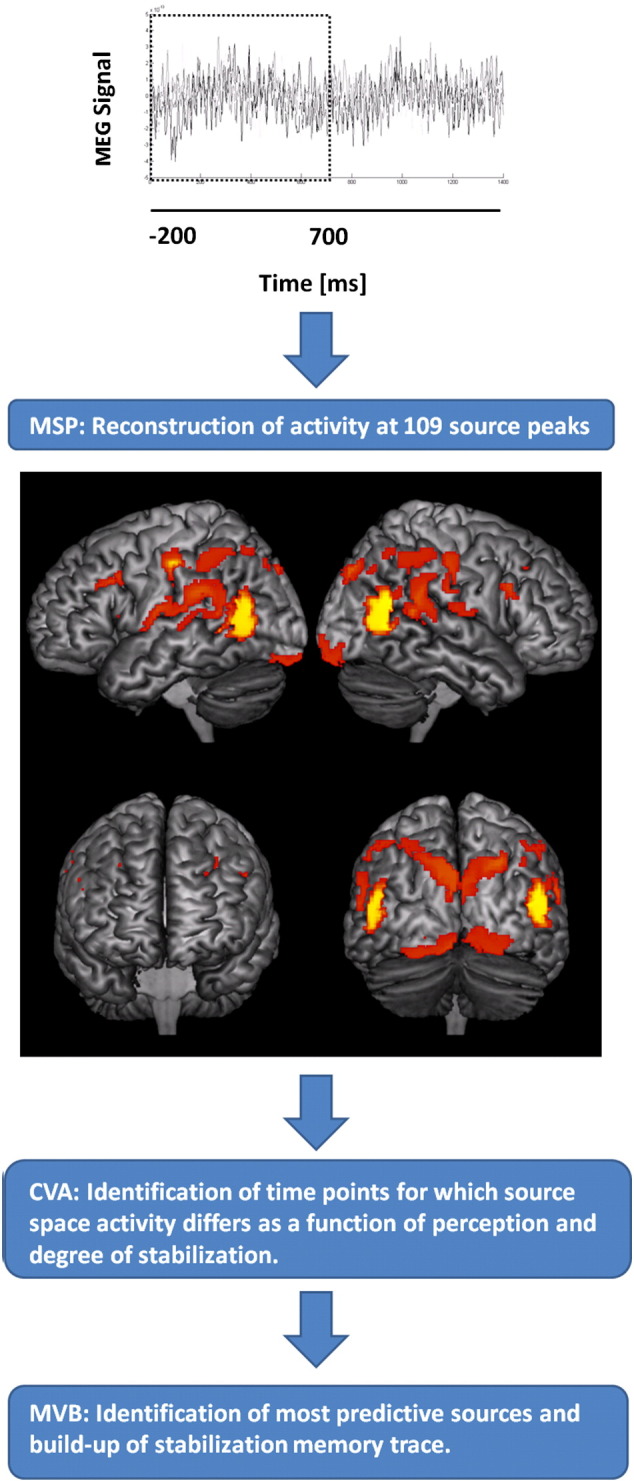
Experimental analyses. Participants’ neural activity during performance of the task was measured with MEG. For analyses, the most active sources during the stimulation period were identified and activity was reconstructed at these sources. The figure shows the most activated sources 0–700 ms after stimulus onset independently of trial type across all participants. All analyses were performed on source reconstructed datasets. Canonical variates analysis (CVA) was performed to identify the time points for which the MEG signal varied as a function of perception (face vs. grating) and as a function of stabilization/destabilization (linear models based on the trial number relative to a perceptual reversal as explained in Fig. 1). Next, Multivariate Bayesian (MVB) models were constructed for each cortical area to compare the relative importance of the areas for perception and stabilization/destabilization. Finally, different MVB models of the stabilization/destabilization memory trace were compared.

A schematic of the basic data analysis is shown in Fig. 2. In the sections below, each analysis step is explained in detail. In brief, first the data were projected into cortical space using the multiple sparse priors algorithm (Friston et al., 2008b, Litvak and Friston, 2008). This gave us approximately 109 sources or regions of interest each with an individual time series. We then used canonical variates analysis (Chatfield and Collins, 1980), CVA, to determine the times at which any linear mixture of sources (regardless of source location) could explain the behavioral response. In order to examine these time periods in more detail we then used a multivariate Bayesian, MVB, scheme (Friston et al., 2008a) to test how well single anatomical regions (each consisting of a subset of the original sources) could explain the behavioral responses.

To clarify our notation, in the following we use the terms “sources”, “features”, and “components”. We use the term ‘component’ to describe typical evoked response temporal peaks of interest (like the M170). A source, in this context, simply refers to a cortical location. A feature is a data reduction device and comprises a linear combination of sources; importantly it is defined without reference to the experimental design. This data reduction increases statistical power by reducing the number of variables in the multivariate test. So for example, for the canonical variates analysis in each trial at each time point, we consider 120 sources that we reduce to 20 features, we then identified linear combinations of these features (canonical vectors) that explained linear combinations (also canonical vectors) of the behavioral data. Classical CVA returns a statistic (in this case Chi squared) per pair of canonical vectors that can be tested against a well-defined null distribution (Chatfield and Collins, 1980). The first canonical vector pair is the combination of features that predicts perception (or stabilization/destabilization) the best, and the second vector pair (which has to be uncorrelated with the first) is the combination of features that explains perception the second best, and so on. If there is only a single behavioral regressor, there is effectively only one canonical vector pair. In this manuscript we only deal with the first canonical vector pair.

#### Source space activity reconstruction

**Fig. 3:**
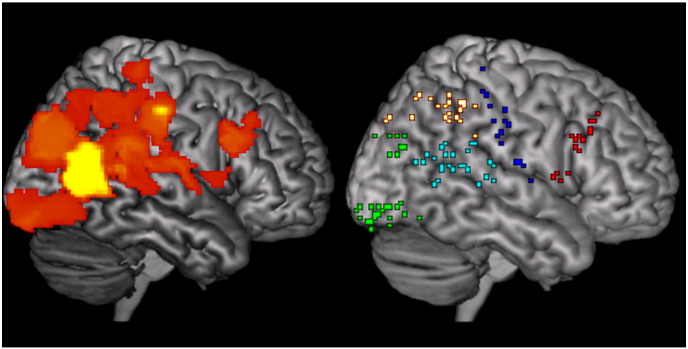
Source peaks for activity reconstruction. Left: Sources for which MEG field strength amplitude across all trials was different from zero at an uncorrected significance threshold of p < 0.05 (shown with infinite search depth). Right: Source peaks at which activity was reconstructed and analyzed. Activity was reconstructed at a source peak if the field strength amplitude across all trials was different from zero at a significance threshold p < 0.05 (uncorrected for multiple comparisons) with a minimum peak-to-peak distance of 3 mm. This liberal criterion for source activation was selected to ensure a large number of sources (109) for subsequent multivariate analyses on which conclusions are based. Sources were grouped according to overall cortical position: Green = occipital lobe, cyan = temporal lobe, hot red/yellow = parietal lobe, blue = precentral/postcentral gyri, red = prefrontal areas. Note that these color codes are unrelated to those used in Fig. 4, Fig. 5, Fig. 8, Fig. 9.

In this section, we project the MEG sensor level data to the brain space. This means that we gain some anatomical specificity and avoid problems with sensor level data which will depend on the head position of an individual. Source analysis was performed within a window from − 200 to 700 ms around stimulus onset using the multiple sparse priors (MSP) algorithm (Friston et al., 2008b) based on all trials (over all conditions) from all eight participants (Litvak and Friston, 2008). We used an inverse normalized canonical brain to provide the structural and a single shell approximation to the inner skull boundary to give the forward model (Nolte, 2003). The MSP algorithm operates by finding the minimum number of patches (least complexity) on a canonical cortical mesh that explain the largest amount of variance (most accuracy) in the MEG data. This tradeoff between complexity and accuracy is optimized through maximization of model evidence in a variational Laplace scheme (Friston et al., 2007). The group level inversion restricts the sources to be the same in all participants, but allows for different activation levels. The analysis identified 109 source peaks activated during stimulus presentation (0–700 ms) at an uncorrected threshold of p < 0.05. This liberal threshold was selected in order to ensure that data from a wide distribution of cortical sources would be included in the analyses below. The activity map and the map of selected source peaks can be seen in Fig. 3. All subsequent analyses were conducted on the (typically > 1300) single trial current density estimates across the 109 cortical sources for each participant.

#### Canonical variates analysis (CVA)

In this section, we want to establish how (if at all) the estimated electrical activity across the cortical surface might predict the behavioral data. CVA was used at a single-participant level to establish the time points for which activity differed significantly as a function of perceptual content, face stabilization, grating stabilization, and stabilization in general. CVA is a generic multivariate framework which subsumes a number of univariate and multivariate tests (like linear regression, ANCOVA, MANOVA etc.). It provides a single statistic (in this case chi square, an approximation to Wilk’s Lambda for reasonable (> 30) degrees of freedom) which describes the ratio of explained to unexplained (co)variance (and therefore becomes equivalent to F for univariate tests). In this case, we wanted to examine if there were any linear combinations of cortical sources that could explain the behavioral data (over trials). For all models, this was performed by extracting 20 orthogonal features using principal components analysis (PCA) and based on all conditions, at a single time point from across the 109 source space estimates per trial (typically N_t_ > 1300 trials). In principle, we could have used all 109 sources as input features, but as many of the source space estimates were highly linearly correlated we chose to reduce this to 20 orthogonal features for computational efficiency and because our region of interest (MVB) analysis below also used around 20 sources per area. We should note, however, that the number of components (in the interval between 8 and 50) had little or no impact on the results as the number of trials was so large in comparison. Canonical correlation was calculated between the N_t_ behavioral measures (e.g. perception of face) and the N_t_ (rows) × 20 (columns) source level estimates separately for each time point (i.e. every 3.33 ms) across the − 200 to 700 ms time window (i.e. this is a multiple regression problem). For Bonferroni correction, we corrected for 21 comparisons reflecting the effective 33 ms smoothness of the 30 Hz low-pass filtered data across the 700 ms of the epoch for which the stimuli were presented. As at each time point we were conducting a single multivariate test (with an analytically well described null distribution) rather than a series of univariate tests there was no need to correct for multiple comparisons over space. These analyses are shown in Fig. 5.

#### Multivariate Bayesian (MVB) model testing

Finally we wanted to look at how cortical activity within specific anatomical regions over trials could be predicted by the perceptual state on previous trials. In order to examine cortical sources where activity varied as a function of perceptual content and stabilization/reversals across participants, we constructed MVB models (Friston et al., 2008a) consisting of subsets of five groups of sources corresponding to different cortical areas (see Fig. 3). The five source groups were selected in order to reduce the total of 109 sources to a meaningful subset based on a division of the cortex into anatomically distinct areas. By restricting the sources used to a particular anatomical area (whilst attempting to explain the same behavioral data) we were able to compute model evidence values showing the relative importance of each anatomical region (in predicting behavior). This meant that now rather than the features being orthogonal mixtures of sources, each source (within the anatomical area) became a feature. We then used a random effects analysis to pool these individual model evidence values over the group (Stephan et al., 2009). This analysis was performed at the peaks in statistical significance identified by the CVA. The significant canonical vectors identified in the CVA typically had a temporal spread 50–80 ms with the peaks of the individual participants occurring at slightly different time points. We therefore computed model evidence at 10 ms steps over each subject over this time range and the resulting model probabilities were averaged (over time) across the entire peak time window. These peak time windows are listed when results are first presented (i.e. in Fig. 6). MVB was also used to examine the stabilization memory trace. We did this by constructing a behavioral response vector in which each trial either contained a function of the number of stimulus presentations since the last perceptual reversal (in the stabilization analysis) or the number of presentations until the next reversal (in the destabilization analysis). Each function was a count of the number of trials since (or until) the previous (next) perceptual reversal with fixed ceiling (between 2 and 10 trials)(see Fig. 7A). Using the data from each region in turn, we then compared the model evidence for each of these different functions (Fig. 8, Fig. 9).

## Results

As expected from previous studies (Leopold et al., 2002, Orbach et al., 1963), blank periods between stimulus presentations of around 800 ms resulted in a high degree of stabilization while nevertheless experiencing occasional perceptual reversals (average probability of stabilization from trial to trial was 87% (SD = 8.8) for face perception and 86% (SD = 13.7) for grating perception). This setup thus allowed us to distinguish the gradual build-up of signals related to stabilization/destabilization from activity specific to the trials associated with a perceptual reversal.

We first examined the correlates of conscious perception during intermittent presentation of binocular rivalry. This allowed us to distinguish stimulus-specific from non-stimulus-specific correlates of stabilization/destabilization.

### Percept-related activity

Based on more than 30 independent EEG studies employing different paradigms, Koivisto and Revonsuo (2010) conclude that visual awareness is correlated with activity often referred to as the Visual Awareness Negativity (VAN). At least 3 MEG experiments have reported activity corresponding to the VAN (Liu et al., 2012, Sandberg et al., 2013, Vanni et al., 1996). The exact time window of the VAN varies slightly between studies but is typically within the 130–320 ms time window (see for instance Busch et al., 2010, Koivisto, 2005). The VAN can be divided temporally into an early and a late part (Koivisto and Revonsuo, 2010), and in some experiments, these two parts of the VAN are observed as separate ERP/ERF components (Fahrenfort et al., 2007, Sandberg et al., 2013). Although authors of various studies interpret their findings slightly differently, most emphasize the importance of one or both of these ERP components in visual awareness. However, although Dehaene and others report that signals around the second VAN component, peaking around 270 ms, correlate with subjective, graded ratings of visibility, they consider temporally later, bimodal responses as the correlates of conscious report (Dehaene et al., 1998, Dehaene et al., 2006, Sergent and Dehaene, 2004, Sergent et al., 2005). Based on these previous findings, we hypothesized that reports of perceptual content during rivalry would correlate with MEG field strength mainly around latencies of the two face-specific peaks in the 130–320 ms interval, the M170 and the P2m, but later components were also considered.

**Fig. 4:**
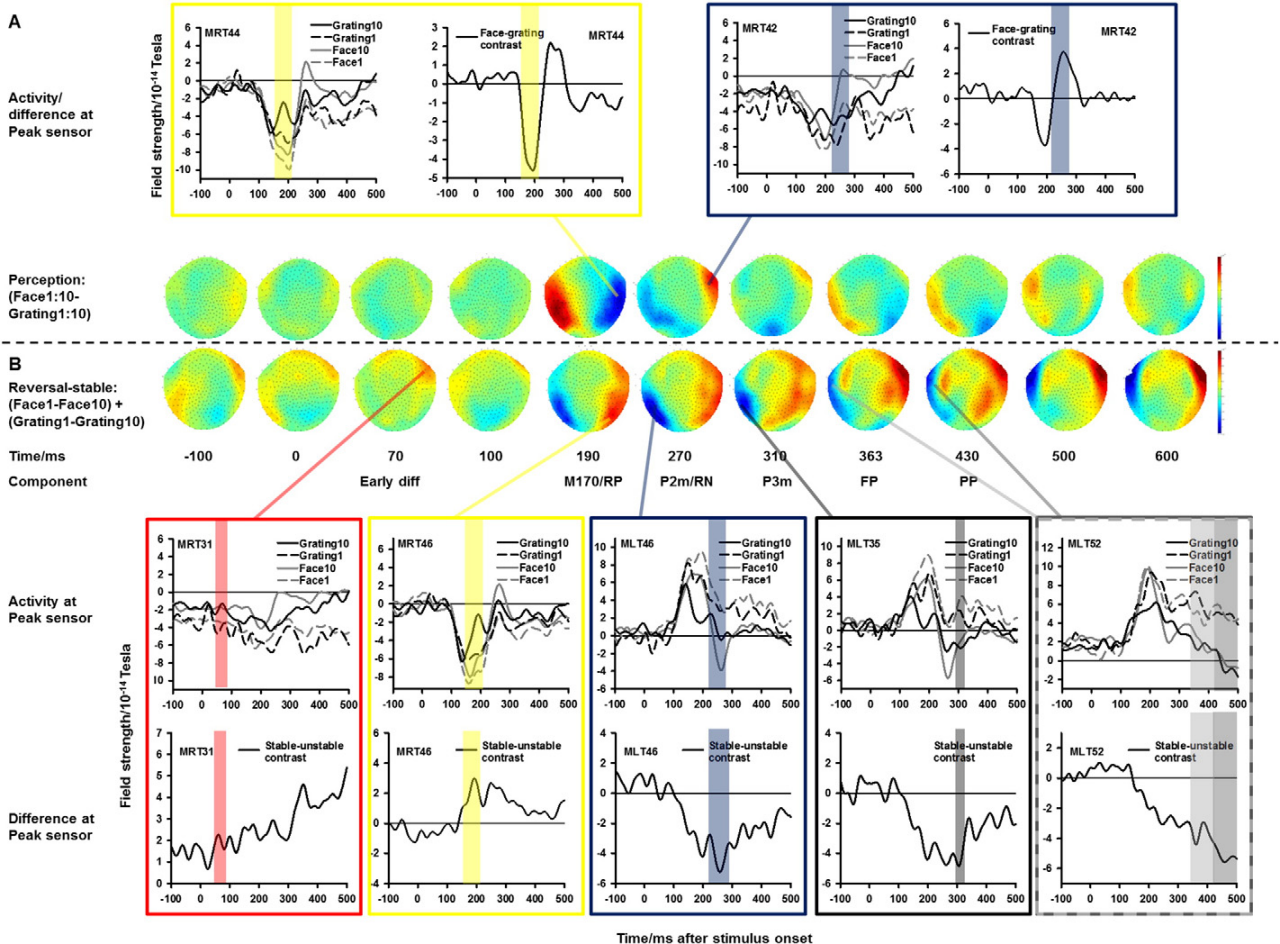
Event-related fields (ERFs) and topographies for illustrative purposes. RP: Reversal positivity. RN: Reversal negativity. FP: Frontopolar positivity. PP: Parietal positivity. A) Topography (bottom) and event-related activity (top) for the face-grating contrast (averages of all Grating 1–10 trials subtracted from averages of all Face 1–10 trials). Large differences in activity as a function of reported perceptual content (face/grating) was found around the M170 (190 ms after stimulus onset) and the P2m (260 ms after stimulus onset). For each component, the ERF graph window on the left plots the ERFs for trials immediately following a reversal (Face 1 and Grating 1) as well as for trials when perception was fully stable (Face 10 and Grating 10). The ERF graph window on the right plots the face-grating contrast using all trials. Note that the difference is present for both types of trials but also that it is larger for fully stable perception. B) Topography (top) and event-related activity (bottom) for the stabilization/reversal related activity. Large differences in activity between trials immediately after a reversal (Face 1/Grating 1) and trials with fully stable perception (Face 10/Grating 10) were found at 70, 190, 260, 310, 363, and 430 ms after stimulus presentation. For each component, the ERF graph window at the top plots the ERFs for all four above-mentioned trial types. The ERF graph window at the bottom plots the stabilization contrast for face (Face 1–Face 10) and grating (Grating 1–Grating 10) trials combined. Please note that these plots are for illustrative purposes only and do not take into account intermediate trials (Face 2–9/Grating 2–9), and neither are multivariate effects considered. Conclusions are based on analyses below. For improved readability, the color codes for the ERF plots are reused in Fig. 5, Fig. 8, Fig. 9. Yellow, for instance, always marks the M170, and the degree of significance of the component is reported in Fig. 5 whereas stabilization vs. reversal model fit is reported in Fig. 8, Fig. 9.

Most previous studies use univariate (primarily ERP) analyses. We have nevertheless previously demonstrated that perceptual content during intermittent binocular rivalry can be predicted highly significantly (with around 80% accuracy) using a combination of sensors (i.e. multivariate analysis) which in univariate analyses perform no better than chance (50%) (Sandberg et al., 2013). For this reason, we used multivariate analyses (CVA and MVB) in the following. However, for optimal comparison with previous studies, we have plotted topographical maps and ERFs in Fig. 4. As seen here, the largest ERF differences between face and grating perception trials were observed around the M170 (at 190 ms) and the P2m (270 ms) as expected. Components at higher latencies were also identified, but the analyses below show that they are much less predictive of conscious perception.

**Fig. 5:**
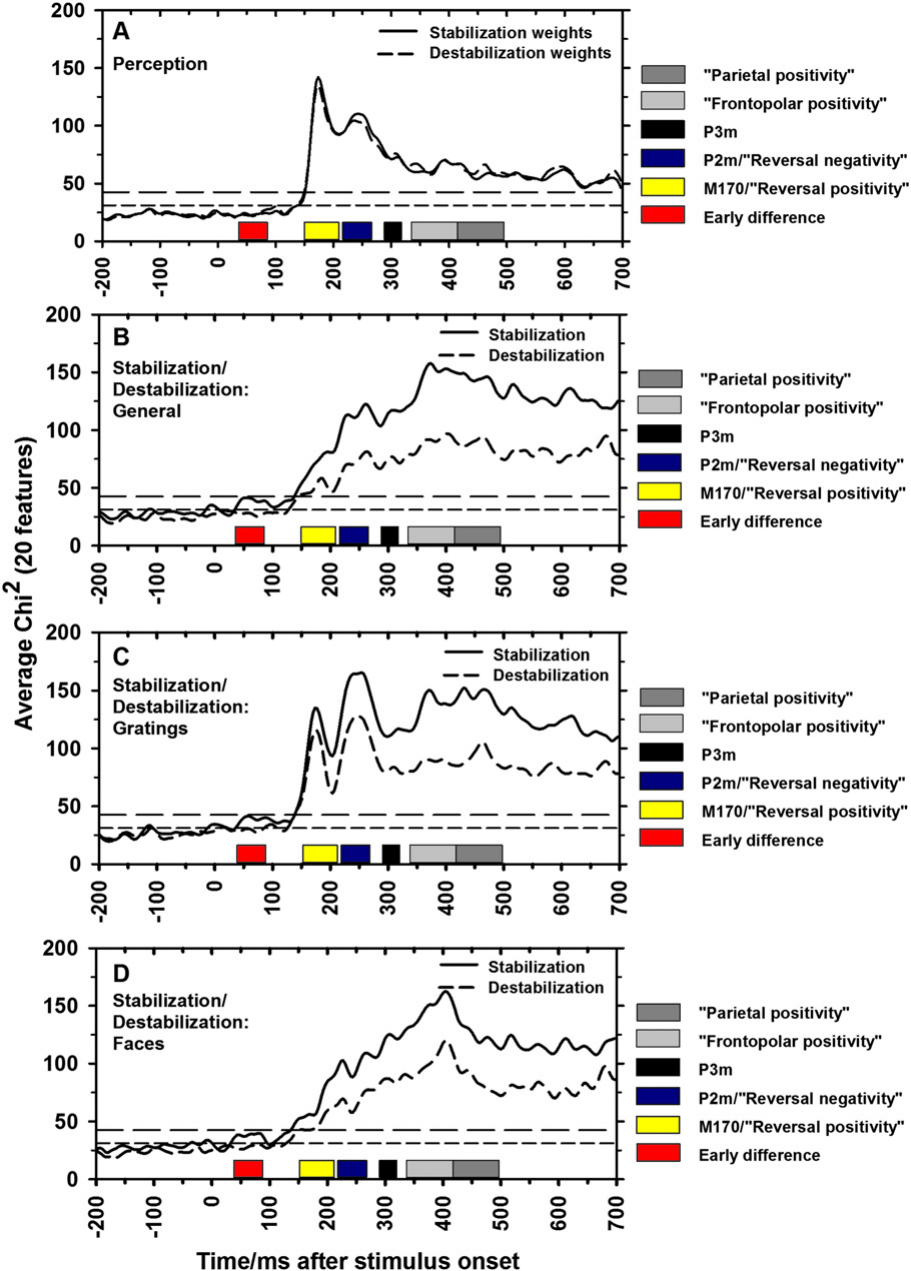
Canonical Variates Analysis (CVA). Average Chi^2^ across participants is plotted as a function of time. Chi^2^ values were obtained using both stabilization and destabilization weights (see Fig. 1 for a description) in separate design matrices. A) Chi^2^ for prediction of perception (Face-Grating contrast using all trials). The highest values were found for perception in the time window of the two face-specific ERF components, the M170 and the P2m. B) Chi^2^ values for stabilization/destabilization in general (i.e. linear models in which both face and grating trials are included and weighted according to the degree of stabilization/destabilization). The dashed line plots CVA evidence based on trial number before a perceptual reversal (i.e. what may be called destabilization), and the solid line plots CVA based on trial number after a perceptual reversal (i.e. stabilization). Note the similar peak latencies for stabilization and destabilization, and note how these differ from those for perception. C–D) Chi^2^ for grating and face perception individually. Note that face stabilization/destabilization values follow those of stabilization in general whereas grating stabilization/destabilization values follow the values of both perception and stabilization. This indicates that stabilization/destabilization of grating perception correlates with modulation of the face-specific M170 and P2m, thus indicating that stabilization correlates with suppression of the non-perceived item. Horizontal medium dashed line: p = 0.05 significance threshold. Horizontal long dashed line: p = 0.05 significance threshold after correcting for multiple comparisons.

**Fig. 6:**
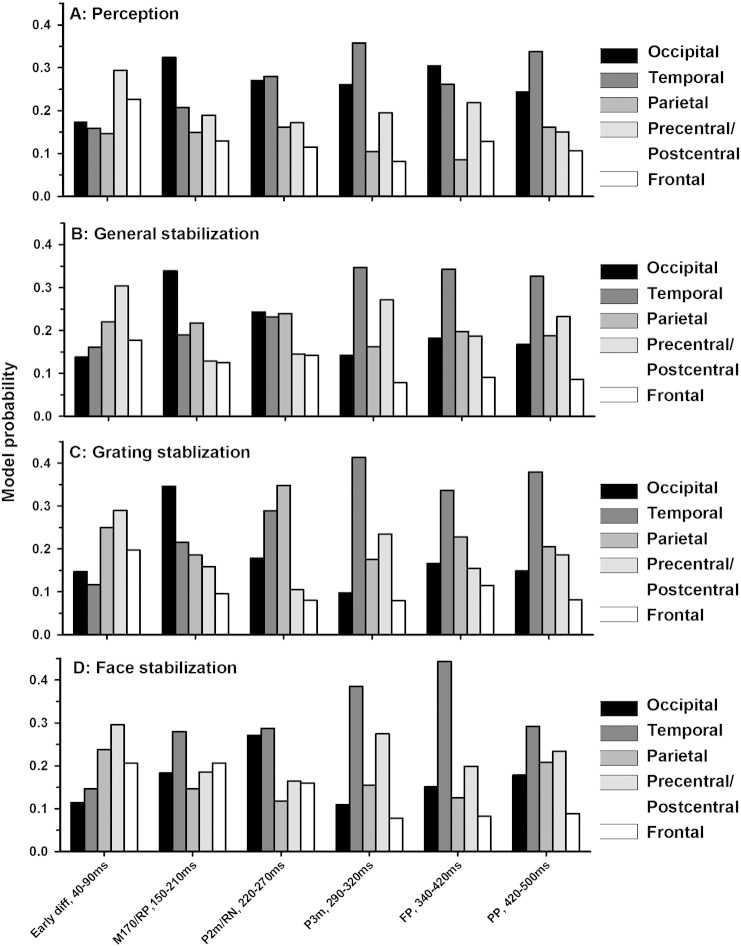
Multivariate Bayesian (MVB) model probabilities. MVB model probabilities that a single cortical area explains the modulation of MEG activity as a function of perception (A) and stabilization (B–D) for all perception and stabilization-related components identified by CVA. Note that perception is primarily explained by extrastriate and to some extent temporal sources whereas parietal sources play a larger role in stabilization at the early components before 290 ms.

*The M170 and the P2m, 150–270 ms*: The main peaks in the canonical correlation between the MEG field strength and perceptual reports were found around the face-specific M170 (150–210 ms) and P2m (220–270 ms) (Fig. 5A). As seen in Fig. 6A, the Bayesian model selection for group studies (Stephan et al., 2009) showed that the models based on occipital and temporal activity were most likely for both components. Not surprisingly, perceptual content was thus generally best explained by ventral stream activity. Components at higher latencies remained above the significance threshold, but were much less predictive.

### Destabilization, stabilization and reversal specific activity

The most consistent ERP correlate of reversals/stabilization is the Reversal Negativity (RN) found around 220–280 ms (Britz and Pitts, 2011, Kornmeier and Bach, 2004, Kornmeier and Bach, 2005, Pitts et al., 2007, Pitts et al., 2009). Reversal/stabilization related modulation of earlier components has nevertheless also been observed, sometimes referred to as Reversal Positivity (RP). The modulations have been observed at around 110–150 ms (Britz and Pitts, 2011, Kornmeier and Bach, 2005, Kornmeier et al., 2011), but in some experiments two distinct modulations have been observed, one around the P1 at around 115 ms and one at the N1 at around 175 ms (Pitts et al., 2007). Similarly, modulation of alpha band activity in a larger time window around 130–200 ms has been observed (Ehm et al., 2011). We thus refer to any effects in this 110–200 ms time window as Reversal Positivity (RP) although the term may have been used to refer to several components in general. Finally, very early correlates (from − 50 to 70 ms) have been observed in some studies (Britz and Pitts, 2011, Britz et al., 2009, Britz et al., 2011).

All these components (the temporally early difference, the RP and the RN) were our main focus of analysis. As seen in Fig. 4, ERF differences between highly stable trials (Face 10/Grating 10) and trials immediately after a reversal (Face + 1/Grating + 1) were found at around the peak times indicated by the existing literature with the earliest (small) difference peaking at around 80 ms, the so-called reversal positivity peaking at 190 ms (the time of the m170), followed by the Reversal Negativity at around 270 ms.

Later correlates were also identified at around 340–420 ms and 420–500 ms corresponding to what has been termed “Frontopolar positivity” (FP) and “Parietal Positivity” (PP) respectively. However, these components have been interpreted as reflecting working memory processes, and recognition/appraisal of a reversal (Britz and Pitts, 2011, Kornmeier and Bach, 2006), or other cognitive processes during (O’Donnell et al., 1988) or following (Isoglu-Alkaç et al., 2000) a perceptual reversal. As they do not appear directly related to the reversal, we report the analyses of these components briefly, but do not provide further in-depth interpretations. We also observed a small separate peak slightly later, at around 300 ms — what we refer to as a P3m. This peak shared sources and trace development with the temporally late peaks and is discussed briefly with those.

Using a single linear model, it is difficult to distinguish activity related to destabilization/stabilization from activity related to reversals in general, as in both cases a difference in activity is observed for trials immediately before/after a reversal compared to intervening trials when perception is stable. The main difference is the temporal extent of the modulations. For reversal-related activity, the modulation is expected only around the reversal; for stabilization-related activity, the modulation is expected to continue across several trials after the reversal. In this section, we do not distinguish between the two. We do, however, distinguish between correlates of stabilization and destabilization; i.e. we refer to the results when using trials *after* a reversal as correlates of stabilization and we refer to the results when using trials *before* a reversal as correlates of destabilization. In the next section, we examine the temporal extent of each component in detail and distinguish between reversal and stabilization/destabilization related activity.

Fig. 5B shows the time points where the MEG signal significantly predicted the degree of destabilization (dashed line) and stabilization (solid line) independently of perception (i.e. at which latencies the MEG signal was linearly modulated in the same way by destabilization/stabilization of faces *and* gratings). Notice that the peaks were observed at or around the latencies of the reversal-related components mentioned above. Fig. 5C–D shows the significance levels for grating and face destabilization/stabilization. Note the similarity of the stabilization and destabilization curves for all analyses. This shows that the latencies for which activity changed across 10 trials *before* a perceptual reversal were almost identical to the latencies for which activity changed across 10 trials *after* a perceptual reversal. Below, we report the CVA evidence and source composition of each component individually.

#### Earliest difference, 40–90 ms

We observed a peak at 40–90 ms. Based on averaged chi^2^ values, the peak was close to significant for grating and general stabilization (p = 0.06, corrected for multiple comparisons). However, at the single subject level the peak was significant for 3 of 8 participants (after Bonferroni correction) (the cumulative probability of observing 3 significant results (at the p = 0.05 level, after correction) out of 8 possible is p = 0.006) (Fig. 5B–C). As shown in Fig. 5B–C, this temporally early peak was mainly explained by precentral/postcentral and parietal sources. Most notable compared to perception-specific activity was the involvement of parietal sources. Interestingly, this peak was present only on trials *following* a reversal, thus indicating a role in the build-up of stabilization. Given the uncertainty about whether this component should be considered significant, we suggest that it is studied separately in experiments with more statistical power to ensure that it is not a false positive.

#### M170/“reversal positivity” (RP), 150–210 ms

This peak was significant for all stabilization and destabilization conditions and peaked at the individual participant level at around 170 ms (Fig. 5B–D). Note that a larger peak was observed for grating stabilization/destabilization than for face stabilization and that this peak followed the pattern of perception evidence. This could indicate that the best correlate of stabilization at this latency was increased suppression of the non-perceived image (that is, stabilized grating perception correlated with suppression of the face-specific M170). As seen in Fig. 6, especially activity in occipital, temporal and parietal regions explained stabilization well at this component. Unlike at most other components, the distribution of source probabilities at the M170/RP was different for face and grating stabilization, with grating stabilization primarily correlating with changes in occipital activity and face stabilization mainly correlating with changes in temporal lobe activity.

#### P2m/“reversal negativity” (RN), 220–270 ms

This peak was observed for general/grating/face stabilization/destabilization (Fig. 5B–D) and again the peak was larger for grating than face stabilization, possibly indicating increased suppression of the non-perceived image as a function of stabilization. As shown in Fig. 6B–D, the main predictor of stabilization/destabilization around this time was activity in occipital, temporal, and parietal areas. As for the M170/RP, the distribution of source probabilities at the P2m/RN was different for face and grating stabilization, with grating stabilization primarily correlating with changes in temporal and parietal activity and face stabilization mainly correlating with changes in occipital and temporal activity. These slightly different profiles for face and grating stabilization, along with the timing of the components, indicate that stabilization at the M170/RP and P2m/RN mainly correlates with modulation of perception-specific activity.

#### P3m and “frontopolar/parietal positivity” (FP/PP), 290–500 ms

These three high latency components were significant for general/grating/face stabilization/destabilization (Fig. 5B–D). Mainly temporal and precentral/postcentral sources were predictive (Fig. 6B–D). The involvement of sensory/motor sources lends support to the earlier claims that these components are not directly related to the stabilization/reversal process.

#### Summary

We found that the amplitude of the MEG signal across several sources predicted the degree of stabilization (ranging from immediately after a reversal to 10 trials after a reversal) and destabilization (ranging from immediately before a reversal to 10 trials before a reversal). Although a very early peak (40–90 ms) was observed for stabilization, the first highly significant peaks were observed around the time range of ERP components in previous studies contrasting reversal and stability trials (the RP and the RN). These two peaks (M170/RP and P2m/RN) were observed at the time points when activity predicted the perceptual content the best, thus indicating that some components related to reversals/stabilization are in fact modulation of percept-specific activity. However, we also identified involvement of parietal sources which were not observed when predicting perceptual content, and these sources thus appeared primarily related to reversals/stabilization. In the section below, we analyze the temporal extent of each component (i.e. across how many trials before/after a reversal it is observed) in order to separate the correlates of destabilization/stabilization from those of perceptual reversals.

### Distinguishing reversal and stabilization related activity

**Fig. 7:**
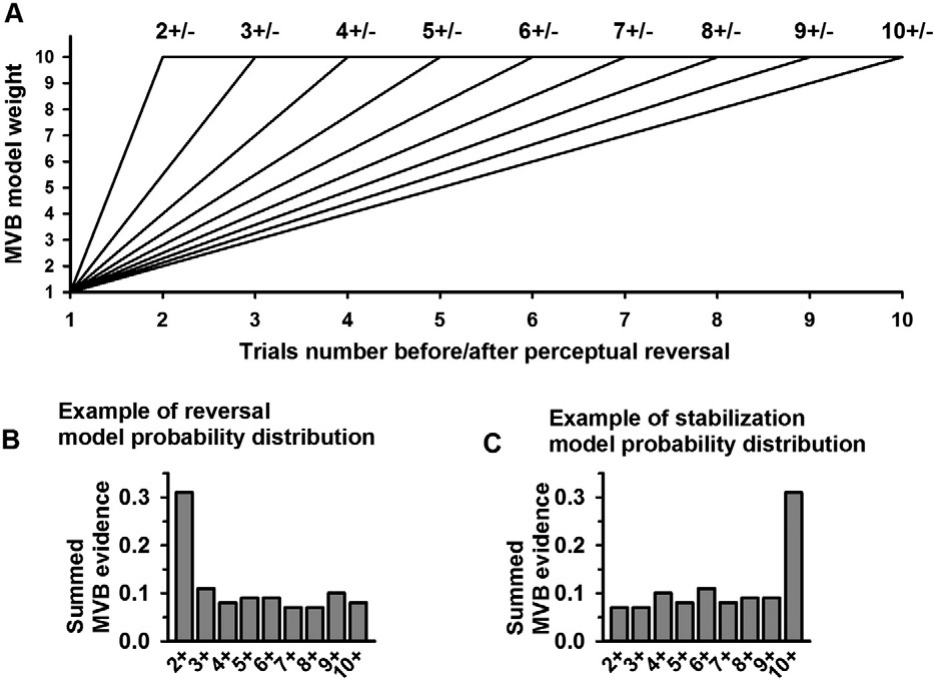
Examples of reversal and stabilization specific MVB model evidence distributions. Nine different linear models were tested for stabilization and destabilization. The model weights are plotted in (A). The models differ in how many trials before/after a perceptual reversal they consider. The model 2 +, for instance, assigns the weight 1 to the trial immediately after a reversal and the weight 10 to any other trial. This model is thus expected to give high evidence levels for components where activity differs mainly between a reversal trial and any other trial. The model 10 +, in contrast, assigns different (increasing) weights to 10 trials following a reversal and is thus expected to give high evidence levels at components for which a gradually evolving stabilization trace is found. (B) Example of model probability when a component is reversal related. High model probability is obtained when comparing trial 1 after a reversal to all other trials, and model probability drops when more distinctions are included in the model. (C) Example of model probability when a component is stabilization related. Model probability is highest when a long stabilization trace of 10 trials is considered in the model.

In order to distinguish the correlates of stabilization/destabilization from those of perceptual reversals, we examined the temporal extent of the memory trace in the cortical areas primarily responsible for the effects. If a component changes gradually across, for instance, 10 trials leading up to a perceptual reversal, the best model would thus be one that assigned monotonically increasing weights to each of the 10 trials leading up to the reversal. The weights for one such possible model can be seen in Fig. 7A, labeled 10 +/−, and the model probability for such a model should thus be higher than for models taking into account fewer trials when slowly developing stabilization trace is present (Fig. 7C). In contrast, if a component is involved primarily in the process of perceptual reversal (i.e. on the trial immediately before or after the reversal) the best model should simply assign a large weight (here 10) to trial 1 after (or before) a reversal and a much smaller (here 1) weight to all other trials. The weights for such a model can be seen in Fig. 7A, labeled 2 +/−. If such a model were correct, including more trials would not improve the model and would result in a worse fit, thus leading to lower model probabilities for such models. An example of this is shown in Fig. 7B.

**Fig. 8:**
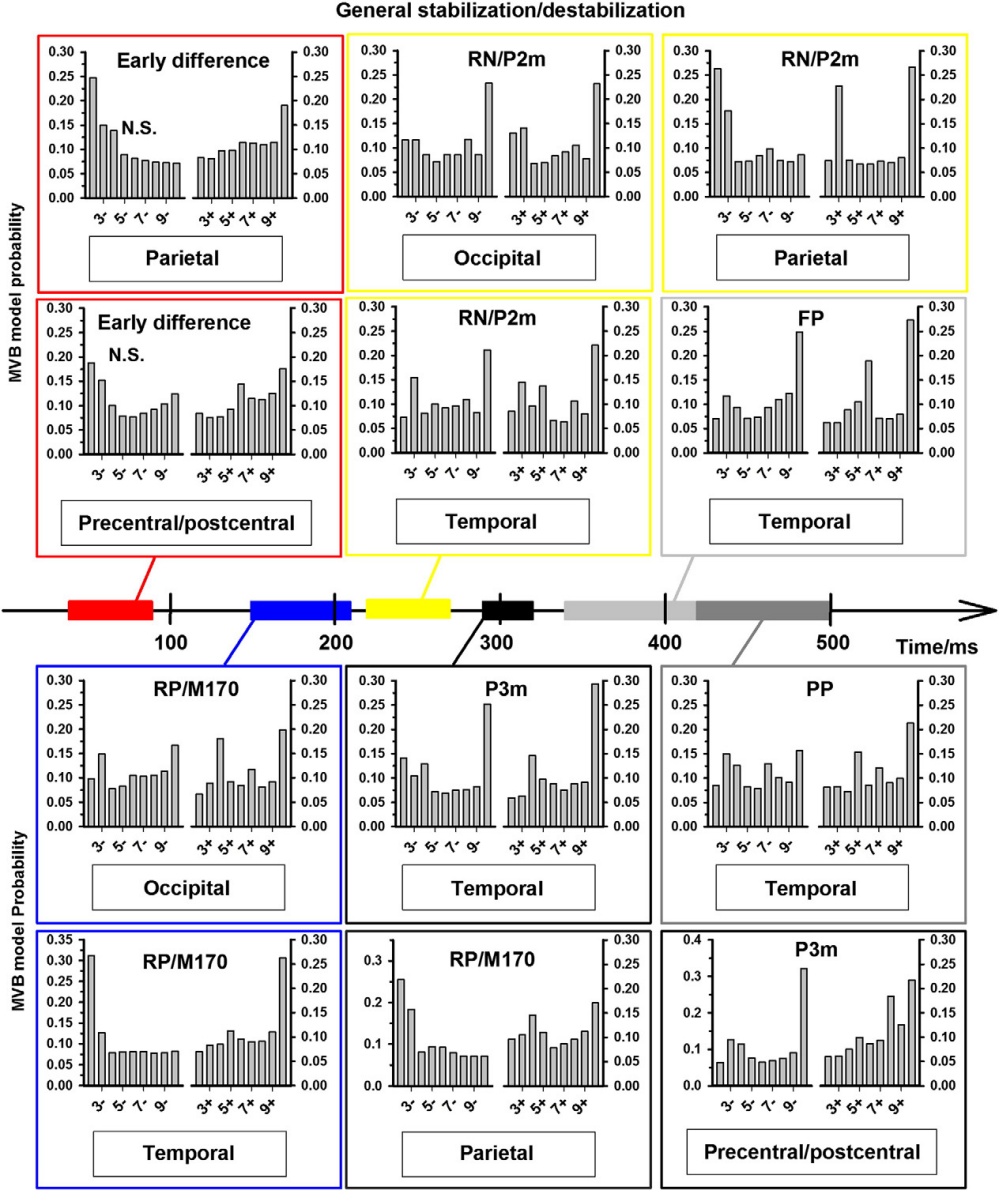
General reversal/(de)stabilization memory trace MVB model probabilities. For all components, stabilization/reversal trace model probabilities were estimated for sources obtaining higher than 20% probability in the analysis reported in Fig. 6B. Note that models taking into account a long stabilization trace (10 trials) typically had high probabilities in occipital/temporal areas whereas reversal related components were localized to activity in parietal sources immediately prior to a reversal (i.e. the 2 − models were most probable).

**Fig. 9:**
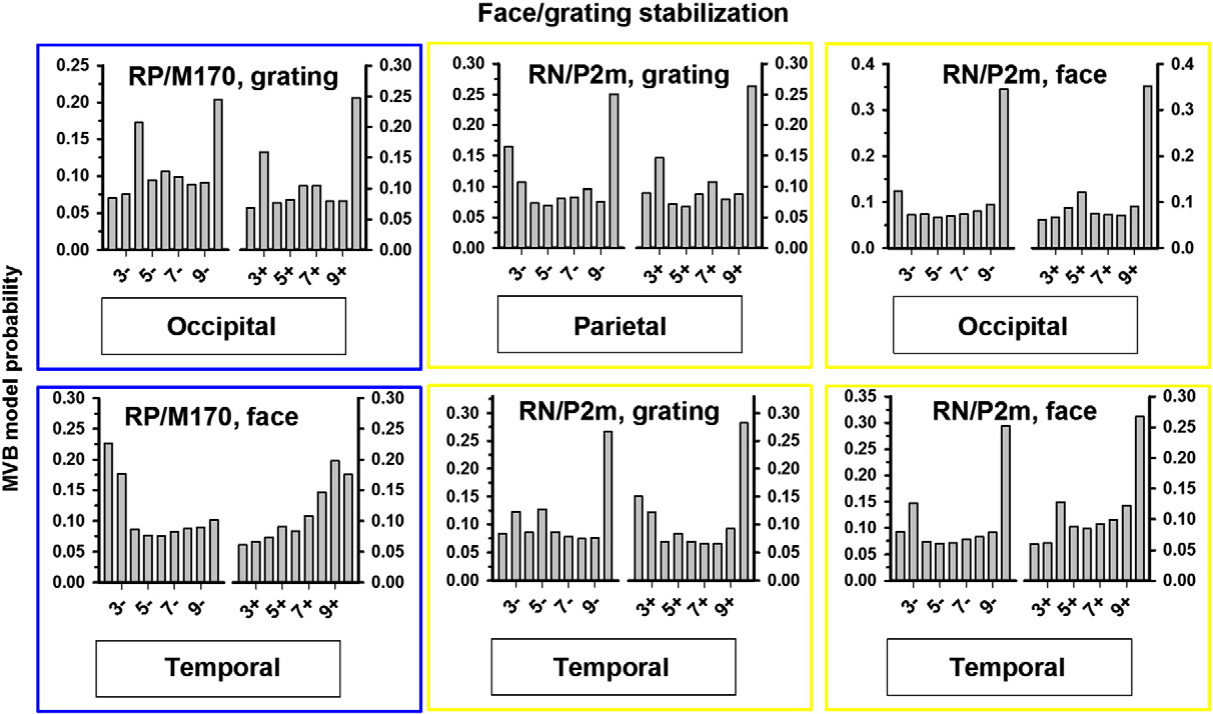
Grating and face reversal/(de)stabilization memory trace MVB model probabilities. For the analysis reported in Fig. 6, face and grating stabilization model probabilities were typically very similar to those for stabilization in general, but not for the M170 and the P2m. For this reason, additional reversal/stabilization trace probabilities were estimated at these two components for face and grating stabilization using the activity at the sources with the highest probabilities in Figs. 6C–D. Results are generally highly consistent with the analysis for general reversal/(de)stabilization.

Stabilization/destabilization trace model evidence was calculated for all models assigned probabilities of greater than 20% in the previous analyses of general stabilization models. The estimated model probabilities of the stabilization/destabilization traces are reported in Fig. 8 by component and source for general stabilization. However, as source probabilities were different between face, grating, and general stabilization for the M170/RP and the P2m/RN, additional trace model probabilities were calculated separately for face and grating stabilization/destabilization for these components at the relevant sources. These results are presented in Fig. 9.

As seen in Fig. 8, Fig. 9, the best model for trials *following* a reversal was the 10 + model for most components/sources, corresponding to a long, robust stabilization trace. Specifically, activity at occipital, temporal and parietal sources around the M170/RP (150–210 ms) and the P2m/RN (220–270 ms) was stabilization related, but also parietal activity at the early difference (40–90 ms) and late temporal (290–500 ms) and precentral/postcentral (290–320 ms) activity was stabilization related. On trials following a reversal, there was no clear evidence for reversal-related activity — only the 3 + model at parietal sites was assigned a high probability, but this was not as high as the probability of the 10 + model. On the trials leading up to a reversal, the 2 − reversal model received the highest probability at parietal sites for the M170/RP and the P2m/RN and at temporal sites for the M170/RP. In contrast, activity at occipital sites around the M170/RP and the P2m/RN was destabilization-related, as was activity at temporal sites for the P2m/RN, the P3 and the FP.

**Fig. 10:**
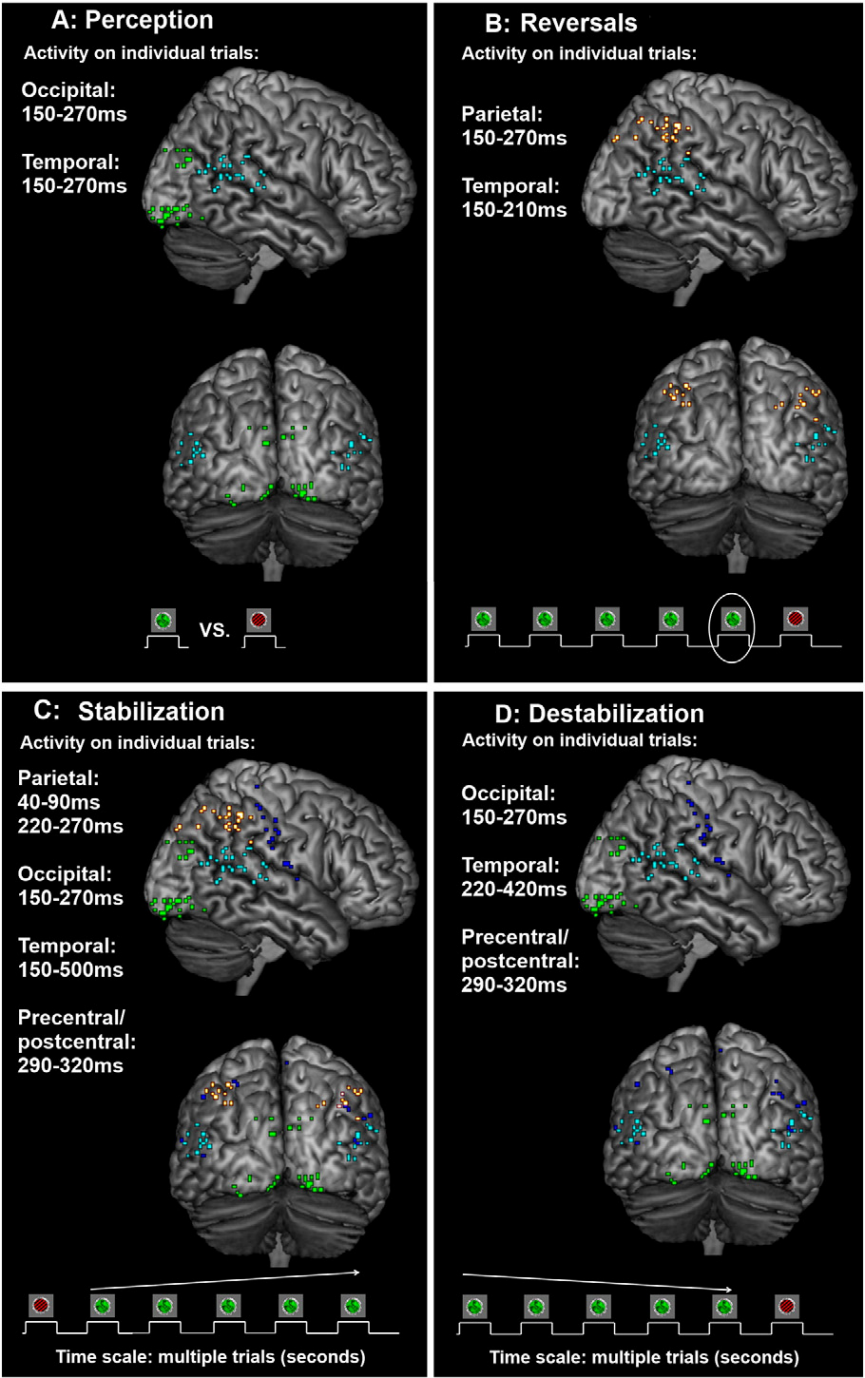
Summary graph. Each graph window (A–D) presents the latencies and sources at which activity correlates with the phenomenon of interest (e.g. perception) relative to stimulus onset. Note that for stabilization/destabilization, the modulation of activity occurs over a larger time scale, i.e. several trials. The main correlate of perception (A) was activity in occipital and temporal cortices in the time window 150–270 ms after stimulus onset. The main correlate of perceptual reversals (B) was parietal activity 150–270 ms and late sensory (temporal) activity at 150–210 ms after stimulus onset on trials immediately prior to a reversal. The main correlates of stabilization (C) and destabilization (D) were modulation of the perception-specific activity across at least 10 trials leading up to and following a perceptual reversal. Activity at temporal locations was also modulated and higher latencies. Additionally, activity at precentral/postcentral sources was modulated briefly around the P2m (290–320 ms). Finally, parietal sources were possibly involved in the buildup of stabilization 40–90 ms and 220–270 ms after stimulus onset on trials following a reversal.

The results are summarized in Fig. 10. Taken together, the results show that there is great overlap in the components and sources involved in conscious perception and destabilization, both involving sensory sources early as well as late in the visual hierarchy. In contrast, only activity at parietal and late sensory (temporal) sources predicted the occurrence of perceptual reversals. The temporally very early predictor of perceptual reversals (i.e. destabilization, observed several trials before a reversal) is thus changes in the activity related to conscious perception whereas the actual reversal is predicted by activity in late sensory (temporal) areas and especially by parietal activity, more closely associated with the attentional network. For stabilization, the results were more mixed as more sources were involved. One interpretation is that stabilization, like destabilization, is related to modulation of activity related to conscious perception (e.g. increased suppression of the non-perceived image) as these, again, were the main sources involved, but the attentional network may be involved in building up the stabilization memory trace. This interpretation is discussed further below.

## Discussion

In the analyses above, we disambiguated the neural correlates of the contents of perception, stabilization/destabilization and perceptual reversals during rivalry through detailed analysis of a large dataset from a small number of participants.

### Perception

We first found that perceptual content correlated mainly with occipital, but also temporal, activity around the M170 and the P2m in the 150–270 ms time window (Fig. 10A). This corresponds very well with the findings of a number of EEG studies (Koivisto and Revonsuo, 2010) as well as a previous analysis of the present dataset using different methods (Sandberg et al., 2013).

One line of EEG/MEG research has used analyses of frequency-tagged bistable perception in sensor space (Srinivasan et al., 1999, Tononi et al., 1998) and source space (Cosmelli et al., 2004) to argue that perception in this case is related to the degree of synchronization between distant brain areas in accordance with information integration theories of consciousness. However, some later studies have found mainly modulation of activity at early visual (occipital) sources during frequency-tagged bistable perception (Kamphuisen et al., 2008, Parkkonen et al., 2008). Furthermore, one of these studies (Kamphuisen et al., 2008) concluded that a limited set of occipital sources could account for the apparent widespread synchronization (i.e. the widespread activity could in fact originate from the same source). This phenomenon is called field spread and is a problem for synchronization measures even at the source level (Schoffelen and Gross, 2009). In accordance with the latter studies, we found evidence that primarily sensory sources predicted perception even though a wide range of sources across the cortex were generally active during binocular rivalry. This supports the notion that visual awareness correlates best with activity in visual areas of the brain, although it is important to emphasize that coherence measures were not employed in our model.

### Stabilization

One fMRI study (De Jong et al., 2012b) and several behavioral studies (Chen and He, 2004, De Jong et al., 2012b, Pearson and Clifford, 2004, Sandberg et al., 2011) have found evidence of stabilization related activity in early and late areas of the visual system as well as in parietal areas whereas other fMRI studies found mainly late sensory and fronto-parietal sources to be important (Schwiedrzik et al., 2012, Sterzer and Rees, 2008). Using EEG, one study found late visual activity around 240–350 ms to be the main correlate of stabilization (Britz and Pitts, 2011). Generally, our results are compatible with these findings and allow for further conclusions.

First, one reason that only one study finds involvement of relatively early visual (occipital) areas may be because these areas, in our study, correlated with stabilization across a much smaller time window than the later sensory areas (150–270 ms vs. 150–600 ms, see Fig. 10C) and at least for fMRI studies this is expected to lead to lower statistical power for low-level visual areas. Second, our results bridge a gap between previous studies based on the BOLD response and studies based on electrophysiological responses with the finding that parietal neuromagnetic signals correlate with stabilization. We further expand the knowledge from the fMRI studies by demonstrating that the parietal cortex may play a role at two different time windows, i.e. immediate after the stimulus-specific signal reaches the cortex (40–90 ms after stimulus onset) and again later during the perception-specific processing of the stimulus (220–270 ms). This indicates that one potential role of the temporally early parietal lobe activity is to bias the incoming visual signal in the direction of the new perceived image after a perceptual reversal, and the component may thus be partially responsible for later, more significant correlates of stabilization.

Third, as shown in previous fMRI studies we find that modulation of perception-related activity is a highly significant correlate of stabilization. We further find that this modulation takes place in the 150–270 ms time window across at least ten trials following a reversal. In other words, stabilization was related to a large difference between the activity related to the perceived and the non-perceived images in the time window and cortical location where activity correlates with the content of consciousness. As the amplitude of particularly the face-specific M170 was much lower for stable than unstable gratings (Grating 1 vs. Grating 10, see Fig. 4B) and as evidence for grating stabilization was higher than for face stabilization at this component, a main correlate of stabilization could be increased suppression of activity related to the non-perceived image. In other words, an increase in stabilization of grating perception led to a reduction of the amplitude of a component typically found to be related to the non-perceived image category. However, not only suppression of the non-perceived image but also increased activity related to the perceived image appeared to be related to stabilization. Both these findings are consistent with a recent behavioural study (De Jong et al., 2012a) demonstrating that exposure to one of two rivalling stimuli prior to rivalry leads to an immediate positive bias towards the previously perceived stimulus at the onset of rivalry (thus indicating a facilitating effect), but also to a subsequent, longer-lasting increase in duration of perception of the alternative stimulus. Changes in the inhibitory capacity of the previously presented stimulus seem the most straightforward explanation for this change in dominance duration of the alternative stimulus as this was modulated without the stimulus having been shown previously although it should be noted that this is an interpretation as MEG does not provide a direct measure of inhibition. In our experiment, we thus interpret the build-up of stabilization to be related to a build-up of modulation of consciousness-specific activity (inhibition and/or facilitation) that was “remembered” and increased from trial to trial in visual areas, possibly assisted by parietal activity.

The interpretation of stabilization as related to gradually increasing inhibition of activity related to the non-perceived image fits well with the recent proposal by Klink et al. (2010) of the involvement of an anti-Hebbian learning mechanism for inhibitory synapses in binocular vision. In this framework, the interocular inhibition during binocular rivalry is remembered across a period of stimulus removal (even when different stimuli are displayed if these do not activate the same cells). In the intermittent presentation paradigm, the blank intervals would thus not interfere with the strength and direction of the inhibition, but would nevertheless decrease adaptation, thus reducing the probability of perceptual instability and allowing for further build-up of the inhibitory connection strength on the following trial.

Finally, we found that the sources we labelled as precentral/postcentral correlated with stabilization 290–320 ms after stimulus onset, and possibly also already 40–90 ms after stimulus onset. This could be taken as evidence of a correlation between stabilization and sensory/motor activity, however other interpretations are also possible. One alternative explanation could be that we are in fact measuring an insula response as this area was included in the model, and at least one fMRI study has found the insula to be involved in stabilization (Schwiedrzik et al., 2012). We suggest that particularly the temporally early component is studied separately in experiments with more statistical power.

### Destabilization

Interestingly, the main correlate of destabilization was changes in perception-specific activity at occipital sources around 150–270 ms as well as at 220–270 ms and slightly higher latencies at temporal sources (Fig. 10D). This finding is consistent with the claim that adaptation of the neurons responsible for inhibiting the suppressed image culminates in perceptual reversals as explained above (Noest et al., 2007, Wilson, 2007). In other words, we interpret the findings of a long destabilization trace as an indication of a slow build-up of adaptation of the neurons responsible for suppressing the activity related to the non-perceived image. As temporal sources were involved during a shorter time window for destabilization than for stabilization, the data are also largely consistent with one fMRI experiment finding that adaptation leads to reversals and that the adaptation process primarily occurs in areas of the occipital lobe, i.e. in the early parts of the cortical visual system (Schwiedrzik et al., 2012). In further agreement with that fMRI study, we found no involvement of parietal sources.

Generally, our data are thus consistent with the notion that while adaptation is slowed by the blank interval, it is nevertheless not prevented entirely, and a gradual build-up of adaptation will lead to a gradual removal of inhibition of the suppressed image. Changes in the stimulus-specific activity in the 150–270 time window across many trials was thus in principle a much earlier predictor of perceptual reversals compared to the parietal activity observed only on trials immediately prior to a reversal (see below). We expand upon the previous fMRI experiment by providing the temporal details of the correlates of stabilization/destabilization, both in terms of the latency (relative to stimulus onset) of components related to stabilization, but also the general extent of the destabilization trace across trials, which has not been examined previously.

### Reversals

In apparent contrast to the models predicting destabilization are findings of fronto-parietal activity during perceptual reversals (Lumer et al., 1998, Sterzer and Kleinschmidt, 2007). Such activity may, however, be more closely related to our findings of activity changes on trials immediately before a reversal.

We identified parietal lobe activity at around 150–270 ms after stimulus onset and temporal lobe activity 150–210 ms after stimulus onset on trials immediately prior to a perceptual reversal (Fig. 10B). This activity corresponded well with the so-called Reversal Positivity (Kornmeier and Bach, 2005, Kornmeier and Bach, 2012) although the latency was slightly delayed compared to some studies, but seemed to fit with a response reported by Pitts et al. (2007) for reversals for Rubin’s vase and Schröder’s staircase as well as with a temporal lobe response also found to be predictive at 180 ms on the trial prior to a reversal (O’Shea et al., 2013). In order to resolve the apparent conflict between theories emphasizing a role of visual areas and theories emphasizing a role of fronto-parietal areas, one fMRI study (Knapen et al., 2011) has claimed that parietal/prefrontal activity around reversals of perception during continuous rivalry is a consequence of an ongoing reversal and not a cause of it as it is present during exposure to monocular displays of mixed perception and appears during transitions of continuous binocular rivalry. Nevertheless, some experimental findings argue against this interpretation and suggest a causal role for frontal and parietal activity. For example, De Graaf et al. (2011) used TMS to demonstrate a causal role of the frontal cortex specifically for voluntary reversals whereas Carmel et al. (2010) and Kanai et al., 2010, Kanai et al., 2011 demonstrate that at least two parietal sites play a causal role in reversals of ambiguous perception. Our parietal model included areas very close to the peak coordinates reported by these previous studies. Additionally, we have previously shown (Sandberg et al., 2013) that reaction times when reporting conscious experience during intermittent BR decrease as a function of stabilization across at least 10 trials before and after a reversal whereas this is not the case when the participant experiences a series of mixed perception. This indicates that perceptual clarity increases across at least 10 trials after a reversal, but parietal activity is only present immediately before and after a reversal. Based on our findings, parietal activity thus appears more closely tied to reversals of perception than to unclear or mixed perception in general.

Overall, our findings may thus be interpreted as support for causal roles for both visual and parietal areas, and in fact, there seems to be little reason that these two explanations should be mutually exclusive. By plotting the long-term development of the destabilization trace, it seems clear that changes in extrastriate visual cortex activity is the earliest predictor of a perceptual reversal, yet the reversal occurs only on the trial at which parietal and late visual (i.e. temporal lobe) sources become active.

### General considerations

One approach that seems able to accommodate our findings is the predictive coding framework. In one predictive coding model of binocular rivalry (Hohwy et al., 2008) late sensory areas predict the activity of lower areas based on a hypothesis of what is being perceived. On the trials leading up to a reversal, destabilization leads to a weaker suppression of the activity related to the non-perceived image, which in turns leads to a larger prediction error. The prediction, for instance, that a grating is present in the stimulated area of the visual field fails to account for an increasing amount of face-specific activity, and eventually the hypothesis must be updated and a reversal initiated.

It should be noted that the present study focused only on activity below 30 Hz. This cutoff was chosen because a previous analysis of the data revealed that gamma band activity (above 30 Hz) did not predict conscious content much above chance (Sandberg et al., 2013). However, more detailed analyses of high frequency activity is still needed to determine the exact role of this activity for conscious perception, stabilization, and reversals.

A long destabilization process as separate from restabilization/disambiguation has been suggested previously by Kornmeier and Bach (2012), who argue that it may start as early as immediately following a reversal. Although they predict that this destabilization process should develop across seconds or even minutes, previous evidence have found mainly parietal activity as a predictor of reversals up to around 1 s prior to report (in the continuous case) or 1 s prior to the onset of the trial on which a new percept is reported (in the intermittent case). This is somewhat surprising as it would be expected that destabilization is linked to, for instance, adaptation, would be detectable mainly in perceptual areas of the occipital/temporal lobes. Our study provides the first clear neuroscientific evidence of a long-term destabilization trace, and this trace is indeed linked to activity changes in occipital/temporal areas. Parietal activity, such as that found prior to reversals in other studies, appears exclusively on the trial immediately to a reversal in our study, and we thus link it more closely to the disambiguation/reversal process than to the long-term destabilization. It could either reflect reversal-related processes directly or simply be a marker of maximal instability as suggested by Kornmeier and Bach (2012).

It should further be noted that we have compared our findings to other studies of bistable perception, but as noted, not all of these have used binocular rivalry — some have used ambiguous figures. These different cases of bistable (or multistable) perception have many aspects in common, but there are also some differences. For instance, the suppression during intermittent binocular rivalry is mainly eye specific (Chen and He, 2004, Pearson and Clifford, 2004, Sandberg et al., 2011) whereas this cannot be the case for ambiguous figures. We therefore emphasize that some of the observed differences between studies could also be attributed to slightly different mechanisms involved.

In the discussion of our findings, we have compared the results to those of studies of continuous presentations of ambiguous stimuli, and although there appears to be great overlap in the areas involved, it may reasonably be questioned whether the mechanisms are the same. Indeed, different mechanisms have been proposed to account for the two phenomena (Noest et al., 2007). Nevertheless, it is important to remember that reversal rates are modulated non-monotonically as a function of inter-stimulus interval (ISI) and that so-called continuous presentation is in fact typically a case of intermittent presentation with a very short ISI (dependent on the frequency of the display cycle and luminance changes across the cycle). For these reasons, it may be entirely possible that the behavioral changes caused by gradual increase in ISI may be accounted for not by the appearance of the “extra mechanism” needed to account for the intermittent case (whether it be increased excitability of the dominant neurons (Wilson, 2007), increased subthreshold elevation of baseline activity for the dominant neurons (Noest et al., 2007), or anti-Hebbian learning), but by a relatively larger impact of this mechanism due to the slower increase of adaptation. If this is true, the intermittent paradigm allows for the study of both mechanisms whereas the non-adaptation mechanism is difficult to study in the continuous case.

Our results may also be compared to those using a slightly different paradigm, first used by Kaernbach and colleagues (Kaernbach et al., 1999). Here, one of the rivalling stimuli is replaced by a stimulus identical to the other stimulus (thus abolishing rivalry and instead resulting in fused perception). When the suppressed image is replaced, no change in awareness is expected (as the participant is already perceiving the image now presented to both eyes), but when the dominant image is replaced, awareness changes to the new fused percept. This allows for study of exogenous changes of awareness during continuous viewing, but time-locked to an event, thus allowing for analyses of event-related responses. Since the perceptual reversal is exogenous, larger similarity to percept specific (rather than reversal specific activity) is to be expected. The activity differences obtained using this paradigm (Roeber et al., 2008, Roeber et al., 2011) are somewhat similar to those observed for intermittent rivalry with evidence of an occipital difference around the P1 found in some rivalry studies (e.g. Pitts et al., 2007) as well as differences around 180 ms and from 250 ms and onwards. The main difference appears to be consistent modulation of the P1 component, which is typically not found to be a consistent predictor of awareness (Sandberg et al., 2013). One explanation for this difference could be that changes in perception are linked to a new stimulus being presented to the dominant eye whereas no change is linked to presentation of a stimulus to the suppressed eye. Presentation to the suppressed eye may cause a slight suppression of all stimulus processing, even temporally very early components that could be precursors for awareness without being directly linked.

Some studies have indicated a larger role of the right hemisphere in perceptual reversals (Britz et al., 2009, Ehm et al., 2011) whereas other studies have found evidence for the involvement of both left and right hemispheres (Britz and Pitts, 2011, Kanai et al., 2010), and one study primarily found evidence for left hemisphere activity being predictive (O’Shea et al., 2013). In our study, there was a tendency for hemispheric lateralization towards the right in the raw data, and indeed a previous analysis of the data showed that right hemisphere sensors were generally slightly more predictive of perceptual content (Sandberg et al., 2013). However, the strength of our current multivariate approach is that it combines information from many features (sources in our case), and reducing the number of features to one half would cause the individual models to be composed of a critically low number of sources (below 15, see for instance Sandberg et al. (2013) and Haynes and Rees (2005)). We propose that the question of hemispheric lateralization as well as inter-hemispheric communication is addressed in future studies, possibly using dynamic causal modelling (DCM) as the analysis paradigm. Such a paradigm could also be used to examine the role of different areas within one of our general areas. For instance, it would be interesting to examine if the proposed different functions of the posterior and anterior parts of the superior parietal lobule (Kanai et al., 2011) could be related to the involvement in both stabilization and perceptual reversals.

In summary, we studied the neural correlates of perceptual content, stability of perception and reversals of perception for the first time in a single experiment. We also examined, for the first time, the changes in neural activity across around 15 s during the build-up of stabilization of binocular rivalry as well as the gradual decay of stabilization (i.e. destabilization) and discovered that the temporal development of these two processes was almost identical. We interpret the findings mechanistically as indicating that the human brain maintains stability of perception by suppressing competing interpretations and that gradual adaptation of the inhibiting cells leads to a state of instability that is eventually resolved by parietal and late-stage visual (temporal) sources.

## Footnotes

1 The impact of high-pass filters of 0.1 Hz and 1 Hz on the data was also examined. Whereas the 1 Hz filter appeared to distort particularly some of the later components slightly, the 0.1 Hz filter led to low-frequency noise (Kappenman and Luck, 2010), which in turn led to a poor source reconstruction. In order to ensure that the selected high-pass filter of 0.5 Hz did not lead to invalid results, subsequent CVA analyses were performed on sensor space data at both 0.1 Hz and 0.5 Hz filtered data and very similar patterns were obtained.
